# Central nervous system penetration and enhancement of temozolomide activity in childhood medulloblastoma models by poly(ADP-ribose) polymerase inhibitor AG-014699

**DOI:** 10.1038/sj.bjc.6605946

**Published:** 2010-10-26

**Authors:** R A Daniel, A L Rozanska, E A Mulligan, Y Drew, H D Thomas, D J Castelbuono, Z Hostomsky, E R Plummer, D A Tweddle, A V Boddy, S C Clifford, N J Curtin

**Affiliations:** 1 Northern Institute for Cancer Research, Newcastle University, Paul O’Gorman Building, Newcastle upon Tyne, NE2 4HH, UK; 2 Pfizer Oncology Inc., La Jolla, CA, USA

**Keywords:** medulloblastoma, PARP, CNS, temozolomide, xenograft

## Abstract

**Background:**

Temozolomide shows activity against medulloblastoma, the most common malignant paediatric brain tumour. Poly(ADP-ribose) polymerase (PARP) inhibitors enhance temozolomide activity in extracranial adult and paediatric human malignancies.

**Methods:**

We assessed the effect of AG-014699, a clinically active PARP inhibitor, on temozolomide-induced growth inhibition in human medulloblastoma models. Pharmacokinetic, pharmacodynamic and toxicity assays were performed in tumour-bearing mice.

**Results:**

Sensitivity to temozolomide *in vitro* was consistent with methylguanine methyltransferase (MGMT) and DNA mismatch repair (MMR) status; MGMT**^+^** MMR**^+^** D384Med cells (temozolomide GI_50_=220 *μ*M), representative of most primary medulloblastomas, were sensitised fourfold by AG-014699; MGMT**^−^** MMR**^+^** D425Med cells were hypersensitive (GI_50_=9 *μ*M) and not sensitised by AG-014699, whereas MGMT**^+^** MMR**^−^** temozolomide-resistant D283Med cells (GI_50_=807 *μ*M) were sensitised 20-fold. In xenograft models, co-administration of AG-014699 produced an increase in temozolomide-induced tumour growth delay in D384Med xenografts. Consistent with the *in vitro* data, temozolomide caused complete tumour regressions of D425Med xenografts, whereas D283Med xenografts were relatively resistant. AG-014699 was not toxic, accumulated and reduced PARP activity ⩾75% in xenograft and brain tissues.

**Conclusion:**

We show for the first time central nervous system penetration and inhibition of brain PARP activity by AG-014699. Taken together with our *in vitro* chemosensitisation and toxicity data, these findings support further evaluation of the clinical potential of AG-014699–temozolomide combinations in intra-cranial malignancies.

Medulloblastoma is the most common malignant paediatric brain tumour and accounts for almost 10% of all childhood cancer deaths. The clinical outcome for patients with medulloblastoma is variable and overall ∼40% of children with medulloblastoma will die of their disease (Pizer and Clifford, 2008, 2009). Current therapies for medulloblastoma comprise maximal surgical resection, craniospinal irradiation and chemotherapy. The use of these combined modalities has led to long-term survival rates of ∼80% in standard risk cases and ∼40–60% in high-risk cases (infants <3 years or metastatic disease at presentation). However, the development of relapsed, drug-resistant disease is common, particularly in high-risk cases (∼50%) and long-term side effects associated with these therapies, including intellectual and neuroendocrine impairment associated with craniospinal irradiation (reviewed in Pizer and Clifford, 2009). New therapeutic approaches, particularly those that may result in improved therapeutic outcome and reduced long-term sequelae, are clearly warranted.

Temozolomide shows significant single-agent activity in adult oligodendrogliomas and high-grade astrocytomas and evidence of activity in pre-clinical medulloblastoma models (Friedman *et al*, 1995; Middlemas *et al*, 2000; Macdonald, 2001; van den Brent *et al*, 2003). Recently, Phase I and II trials of temozolomide, using a variety of schedules, have been undertaken in paediatric brain tumour patients, including medulloblastoma (reviewed in Barone *et al*, 2006). The maximum-tolerated dose and dose-limiting toxicities in paediatric patients appear to be similar to those in adults, and encouraging responses have been observed against medulloblastoma (Baruchel *et al*, 2006; Nicholson *et al*, 2007; Wang *et al*, 2009), supporting further investigation of temozolomide in this disease.

DNA damage signalling and repair pathways are novel and promising targets for improved cancer therapy, particularly with regard to radio- and chemosensitisation. Genomic instability, which often results from DNA damage signalling and repair defects, is a common characteristic of cancer. Such defects can render the cancer cell more dependent on complementary signalling and repair pathways, which are often upregulated. These upregulated pathways can compromise the efficacy of DNA-damaging anticancer agents and represent a novel therapeutic target to specifically sensitise cancer cells (reviewed in Curtin, 2007). Resistance to temozolomide therapy has been associated with high levels of methylguanine methyltransferase (MGMT) and defects in mismatch repair (MMR) (Curtin *et al*, 2004). In adult glioma patients, sensitivity to temozolomide is associated with methylation of the *MGMT* promoter (Hegi *et al*, 2005). Attempts to modulate temozolomide resistance through inhibition of MGMT with co-administration of O^6^-benzylguanine have not lived up to their pre-clinical promise in adult brain tumour patients (reviewed in Curtin, 2007). This approach has also been tested in the Phase I setting in children, but the numbers are too small to determine if success is likely to be greater (Broniscer *et al*, 2007). In the pre-clinical setting, defects in DNA MMR are associated with resistance to temozolomide, which is not overcome by MGMT inhibition (Curtin *et al*, 2004; Cheng *et al*, 2005). In the Phase I O^6^-benzylguanine combination study, microsatellite instability, an indication of defective MMR, was observed in four of the six medulloblastoma patients. The MGMT status of primary medulloblastomas is controversial, with variable estimates in the literature, although in general MGMT defects appear relatively uncommon (reviewed in Lindsey *et al*, 2005). Similarly, in other studies, MMR defects occur only in a small subset of medulloblastomas (Viana-Pereira *et al*, 2009).

An alternative strategy to enhance temozolomide activity is to inhibit poly(ADP-ribose) polymerase-1 and -2 (PARP-1 and PARP-2). These enzymes are activated by DNA single- and double-strand breaks and promote their repair through the relaxation of chromatin and recruitment of other repair proteins. We have previously shown that PARP inhibitors can restore temozolomide sensitivity to MMR-defective cells (Curtin *et al*, 2004), and sensitise tumour cells and xenografts, including models competent for MMR and MGMT, to temozolomide (Calabrese *et al*, 2004). The first PARP inhibitor to enter clinical trial for cancer therapy was AG-014699, in combination with temozolomide (Plummer *et al*, 2008), which was selected on the basis of pre-clinical data in models of adult human malignancies (Thomas *et al*, 2007). AG-014699 has favourable pharmacokinetics and causes profound and sustained inhibition of PARP activity in surrogate normal tissues (peripheral blood lymphocytes) and tumours (Plummer *et al*, 2008). In the Phase II setting, AG-014699 doubled the reported response rate to temozolomide (Plummer *et al*, 2006). We have recently been investigating the potential of AG-014699 in models of paediatric malignancies and found that it increased the efficacy of temozolomide and topotecan in neuroblastoma cells and xenografts (Daniel *et al*, 2009).

We wished to investigate if AG-014699 could be beneficial for the treatment of intra-cranial tumours, using medulloblastoma as a model. Central nervous system (CNS) tumours can be more difficult to treat because of the blood–brain barrier (BBB) that limits drug uptake into CNS tissues. The BBB is a physical and biochemical impediment to the transport of drugs into the CNS by virtue of highly impenetrable vascular endothelial cells and an abundance of drug efflux pumps (reviewed in Deeken and Loscher, 2007). In general, for good CNS penetration, drugs should have few hydrogen bond donors and positive charges, lower polar surfaces, reduced flexibility and be <400 Da in size (Deeken and Loscher, 2007). AG-014699 is the phosphate salt of the active compound AG-014447, which has a molecular weight of 323 Da, p*K*_a_ of 9.6 and a *c* log *D* of 0.7 (Thomas *et al*, 2007). It is likely to exist as a protonated amine at physiological pH and has some polar functionalities that may not favour CNS penetration. The BBB has been hypothesised to be less intact in primary and metastatic brain tumours, at least in the case of large tumours, in which the physiology of the endothelial cells is different from the rest of the brain (reviewed in Daniel *et al*, 2009). However, a number of other PARP inhibitors, including ones with similar structure to AG-014447, have been shown to penetrate and have biological activity in the brain in pre-clinical models of adult malignancies (Tentori *et al*, 2003; Hattori *et al*, 2004; Donawho *et al*, 2007). Recent clinical trials of the PARP inhibitor, olaparib, showed evidence of regression in a brain metastasis in one patient (Fong *et al*, 2009). Temozolomide shows good CNS penetration, and uptake into human gliomas was higher than the surrounding normal brain, owing to breakdown of the BBB and possibly tumour-induced angiogenesis (Rosso *et al*, 2009). Furthermore, radiotherapy can also disrupt the BBB (reviewed in Deeken and Loscher, 2007). However, it may be hypothesised that, for smaller tumours including the CNS metastatic deposits commonly associated with medulloblastoma relapse, the BBB may still be intact. It is therefore important to determine if AG-014699 can penetrate CNS tissues.

We report here the pre-clinical assessment of AG-014699, *in vitro* and *in vivo* in human medulloblastoma models. Our data show the distribution of AG-014699 and inhibition of PARP in CNS tissue for the first time and show the clear potential of PARP inhibitors in combination with temozolomide for the improved therapy of medulloblastoma.

## Materials and methods

### Materials

Temozolomide was a gift from Cancer Research UK (London, UK), and AG-014699 (PO_4_ salt of AG-014447, now also called PF-01367338) was a gift from Pfizer Oncology (La Jolla, CA, USA). Temozolomide was dissolved in dimethyl sulphoxide (DMSO) before addition to cell cultures at a final concentration of 0.5% (v v^−1^) DMSO. For *in vivo* evaluation, temozolomide was dissolved in saline immediately before administration. 10H mouse monoclonal antibody to ADP-ribose polymers was a kind gift from Dr Alexander Burkle (University of Konstanz, Konstanz, Germany). Other chemicals and reagents were obtained from Sigma (Poole, UK), unless otherwise stated.

### Cell lines and culture

Three medulloblastoma cell lines were selected for study. D384Med and D425Med were kind gifts from Dr D Bigner (Duke University, Durham, NC, USA). D283Med was obtained from the American Type Culture Collection (Manassas, VA, USA). Published cell line karyotypes and genetic features were confirmed in each cell line before use; all three lines harboured genetic lesions consistent with primary medulloblastomas (Langdon *et al*, 2006). All three medulloblastoma cell lines were maintained using standard methods, in Dulbecco's modified Eagle's medium containing 20% fetal calf serum (Life Technologies, Paisley, UK), and were confirmed to be mycoplasma free.

### Cell line protein expression

Protein lysates were prepared from each cell line using standard methods and examined for the presence of the PARP-1 protein (H-250 anti-PARP-1 antibody; Santa Cruz Biotechnology, Heidelberg, Germany) and *α*-tubulin control using western blot analysis (50 *μ*g protein loaded per well). Lysates were also blotted for cell line expression of MGMT and the MMR proteins MLH1, MSH2, MSH3, MSH6 and PMS2 (all mouse monoclonal antibodies; BD Biosciences Pharmingen, Oxford, UK).

### Inhibition of cellular PARP activity by AG-014699

Inhibition of PARP activity in 5000 exponentially growing D283Med cells was measured following treatment with a range of AG-014699 concentrations (0–1 *μ*M), in comparison with DMSO-only controls. Maximally stimulated PARP activity was measured in replicate samples (*n*⩾3) of permeabilised cells by immunological detection of the amount of poly(ADP-ribose) (PAR) formed, using 10H anti-PAR antibody, during a 6-min incubation with NAD^+^ and oligonucleotide (substrate and activator) by reference to a PAR (Biomol Research Labs, Plymouth, PA, USA) standard curve using a GCLP-validated assay described previously (Plummer *et al*, 2005).

### *In vitro* growth inhibition and cytotoxicity assays

Cell growth inhibition was estimated in exponentially growing D425Med, D283Med and D384Med cells in 96-well plates. Seeding densities of 1 × 10^3^, 3 × 10^3^ and 3 × 10^3^ cells, respectively, ensured exponential growth for the duration of the experiment. At 24 h (D384Med) or 48 h (D283Med and D425Med) after seeding, cells were exposed to varying concentrations of temozolomide, as described in the Results, in the presence or absence of 0.4 *μ*M AG-014699, a concentration previously shown to enhance temozolomide cytotoxicity in adult tumour cell lines (Thomas *et al*, 2007). After 3 days (D425Med and D384Med) or 5 days (D283Med) of culture (equivalent to approximately three cell divisions), the cell viability was quantified using an XTT cell proliferation kit assay (Roche, Mannheim, Germany), according to the manufacturer's instructions. Cell growth was expressed as a percentage in relation to DMSO or 0.4 *μ*M AG-014699-alone controls. The concentration of temozolomide, alone or in combination with AG-014699, that inhibited growth by 50% (GI_50_) was calculated from computer-generated curves (GraphPad Software, San Diego, CA, USA). The potentiation factor_50_ (PF_50_) is defined as the ratio of the GI_50_ of temozolomide in the presence of AG-014699 to the GI_50_ of temozolomide alone. All data were from at least three independent experiments.

### Establishment of D425Med, D283Med and D384Med tumour xenografts

All of the *in vivo* experiments were reviewed and approved by the relevant institutional animal welfare committees, and performed according to national law. Female athymic nude mice (CD1 *nu/nu*; Charles River, Margate, UK) used for anti-tumour studies were maintained and handled in isolators under specific pathogen-free conditions. D425Med, D283Med and D384Med (1 × 10^7^ exponentially growing cells per mouse, harvested and implanted in growth media) xenografts were established by sub-cutaneous implantation into CD-1 nude mice. Before use in experiments, xenograft establishment was defined as when two-dimensional calliper measurements of tumours reached approximately 5 × 5 mm^2^. Treatment was initiated when sufficient number of mice had established tumours to allow randomisation into treatment groups: day 17 for D425Med, day 26 for D384Med and day 32 for D283Med. Tumour-bearing mice were killed 100 days after the start of treatment or when two dimensions of a tumour xenograft reached 10 mm or one dimension reached 15 mm, whichever was the earliest.

### AG-014699 pharmacokinetics and pharmacodynamics in mouse plasma, brain and D283Med tumour xenografts

One or four daily doses of PARP inhibitor AG-014699 (1 mg kg^−1^ intraperitoneally (i.p.)) were given to CD-1 nude mice bearing established D283Med xenografts. At 0.5, 2, 6 and 24 h after the initial or fourth daily dose of AG-014699, three animals per time point were bled by cardiac puncture under general anaesthesia, and then killed. Plasma was separated from the blood samples using standard methods and stored at −80°C. The brains and tumours were removed, snap frozen in liquid nitrogen and stored at −80°C before analysis. Blood, tumour and brain tissue were removed from three untreated control animals and processed in the same way.

### AG-014699 distribution in plasma, brain and tumour xenograft samples

AG-014699 is the phosphate salt of AG-014447 and rapidly liberates the parent drug following injection. The concentration of AG-014447 was determined in plasma and tissue homogenates (1 : 4 (w v^−1^) in PBS) of brain and tumour after protein precipitation with acetonitrile by liquid chromatography/mass spectrometry/mass spectrometry using a turbo ion spray interface and multiple reaction monitoring in the positive ion mode (API 4000; Applied Biosystems, Warrington, Cheshire, UK) and a deuterated internal standard as described previously (Plummer *et al*, 2008). The lower limit of quantitation was 1 ng ml^−1^. Plasma pharmacokinetics of AG-014447 were analysed using a non-compartmental approach in WinNonlin version 5.2.

### Pharmacodynamics in brain and tumour tissue: PARP-1 activity assays

PARP activity was determined in homogenates of subcutaneous D283Med xenografts and brain tissue (see above). Maximally stimulated PARP activity was measured in replicate samples (*n*⩾3) of a 1 : 1000 dilution of the homogenate as described for permeabilised D283Med cells (see above). Data were calculated as pmol PAR per mg protein by reference to the PAR standard curve and protein content of the sample and expressed as a percentage of the corresponding tissue from the saline-treated control animals. The mean PARP activity in xenograft and brain samples taken at each time point was expressed as a percentage of the mean PARP activity of control xenografts from untreated mice (*n*=3).

### Tumour growth inhibition *in vivo*

CD-1 nude mice bearing palpable, established subcutaneous D425Med, D283Med and D384Med xenografts were treated with normal saline (control animals), temozolomide (68 mg kg^−1^ *per os* (p.o.)) or AG-014699 (1 mg kg^−1^ i.p.) alone or in combination, daily for 5 days (five mice per group). For drug combinations, AG-014699 was administered immediately after administering temozolomide. Tumour volumes, determined from two-dimensional calliper measurements and the equation *a*^2^ × *b*/2 (where *a* is the length and *b* is the width of the tumour), were monitored for the experimental period (up to 100 days), and are presented for each group of mice as median relative tumour volume (RTV) values. Relative tumour volume 1 is the tumour volume on the initial day of treatment (day 0), and RTV4 is the tumour volume four times that on the initial day of treatment. Tumour growth delay (TGD) is defined as the time to RTV4 in drug-treated mice compared with the time to RTV4 in control (vehicle alone) mice. Median tumour volume is shown, rather than the mean, as this is generally accepted as the most statistically reliable representation of the average growth rate of tumours in a small group of mice, if a normal distribution of tumour volumes cannot be assumed. Tumour growth delay was calculated as: median time to RTV4 in treated group−median time to RTV4 in control group. Percentage enhancement was calculated as 100 × (TGD TMZ+AG-014699/TGD TMZ alone)−100 (Calabrese *et al*, 2004).

## Results

### AG-014699 and PARP-1 inhibition in permeabilised medulloblastoma cells

AG-014699 is a potent inhibitor of purified full-length human PARP-1 (Thomas *et al*, 2007). We assayed PARP-1 activity in permeabilised D283Med cells after AG-014699 treatment. AG-014699, at concentrations of 0.1, 0.4 and 1 *μ*M, inhibited PARP-1 activity by 81.1, 96.8 and 97.1%, respectively. All previous cellular chemosensitisation studies with this class of PARP inhibitor, including AG-014699 have used a concentration of 0.4 *μ*M (Calabrese *et al*, 2004; Thomas *et al*, 2007). As this concentration also caused almost total inhibition of the PARP activity in medulloblastoma cells, we used a concentration of 0.4 *μ*M AG-014699 to study the chemosensitisation of temozolomide, enabling comparison with previous data.

### Cell line expression of inhibition target and DNA repair pathway proteins

**Figure 1:**
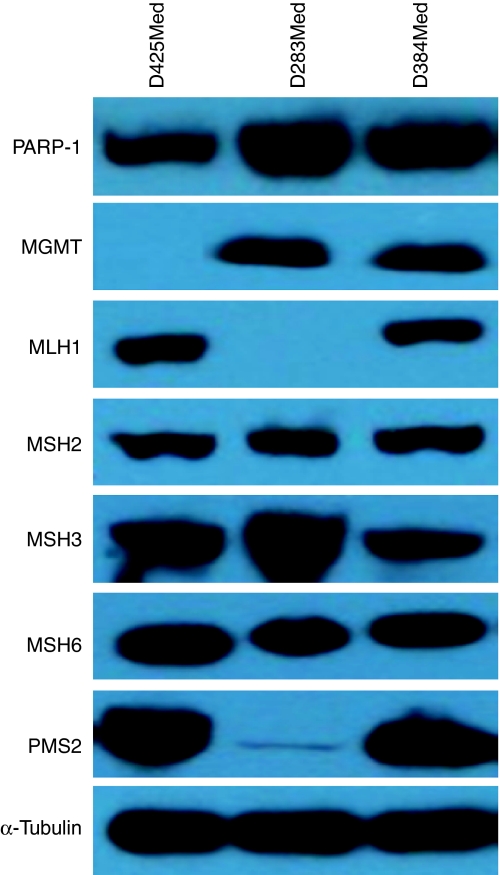
Protein expression levels of PARP-1 and proteins related to temozolomide sensitivity/resistance in medulloblastoma cell lines. Expression levels of *α*-tubulin (control) are also shown. Protein expression was determined for 50 *μ*g of whole-cell lysate by western blot analysis and visualised by chemoluminescence. D425 cells have no detectable MGMT and D283 cells have no detectable MLH-1 and minimal PMS2 consistent with MGMT and mismatch repair deficiency, respectively, whereas D384 cells are proficient for both.

All three cell lines selected for study expressed PARP-1 by western blotting (Figure 1). D384Med also clearly expressed MGMT and the MMR proteins MLH1, MSH2, MSH3, MSH6 and PMS2, indicating competence in these pathways implicated in temozolomide sensitivity. However, two of the cell lines were found to be compromised in the expression of some of these proteins. D425Med lacked MGMT and D283Med was deficient in MLH1 and had barely detectable PMS2, indicating MMR dysfunction.

### Potentiation of temozolomide induced growth inhibition by AG-014699

**Figure 2:**
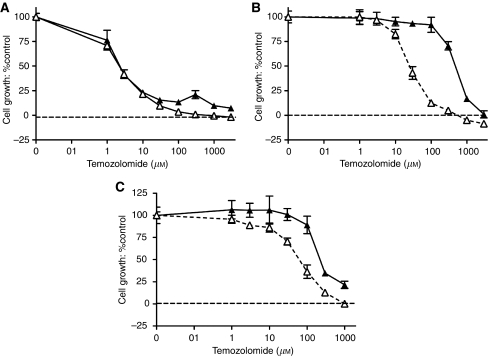
Chemosensitisation to TMZ by AG-014699 *in vitro* in medulloblastoma cell lines. (**A**–**C**) Growth inhibition of D425Med, D283Med and D384Med cells, determined by XTT assay following a 6-day exposure to TMZ either alone (solid triangles) or with 0.4 *μ*M AG-014699 (open triangles). Data are shown normalised to 0.5% DMSO or 0.4 *μ*M AG-014699 controls, respectively. A single representative replicate for TMZ±0.4 *μ*M AG-014699 in D425Med (**A**), D283Med (**B**) and D384Med (**C**) cells (±95% confidence intervals (CI)) is shown.

We measured the growth of cells exposed to increasing concentrations of temozolomide alone or in combination with 0.4 *μ*M AG-014699 continuously, over a period of three cell doublings. Representative growth inhibition curves of D425Med (Figure 2A), D283Med (Figure 2B) and D384Med (Figure 2C) are shown. Pooled GI_50_ data for temozolomide with and without AG-014699 from at least three independent experiments for each of the three cell lines are shown in Table 1. AG-014699 alone was not growth inhibitory at the concentration used (0.4 *μ*M) (data not shown). Temozolomide alone caused a concentration-dependent inhibition of growth in all three cell lines. Cell lines exhibited variable levels of sensitivity to temozolomide alone. The MGMT-deficient D425Med cells were hypersensitive to temozolomide, and the MMR-defective D283Med cells were nearly 100 times less sensitive, as expected. The D384Med cells, which were proficient in both pathways, displayed intermediate sensitivity. There was also considerable variation in the degree of chemosensitisation by AG-014699 between the cells, with almost 20-fold sensitisation of the MMR-defective, D283Med, cells compared with >3-fold sensitisation in the MMR- and MGMT-competent D384Med cells, but no sensitisation of the MGMT-deficient, D425Med, cells.

### AG-014699 levels in plasma, brain tissue and D283Med tumour xenografts

A dose of 1 mg kg^−1^ AG-014699 daily five times has previously been shown to be non-toxic and sufficient for profound chemosensitisation of human colon and neuroblastoma cancer xenografts to temozolomide (Thomas *et al*, 2007; Daniel *et al*, 2009). Therefore, we selected this dose to investigate chemosensitisation of medulloblastoma xenografts. Before undertaking these studies, we determined the distribution of the PARP inhibitor in the plasma, and to brain and tumour tissue, following a single dose and four daily doses of 1 mg AG-014699 per kg i.p. in mice bearing D283Med sub-cutaneous xenografts. We measured concentrations of AG-014447 at either 0.5, 2, 6 or 24 h after dosing (three mice per time point).

**Figure 3:**
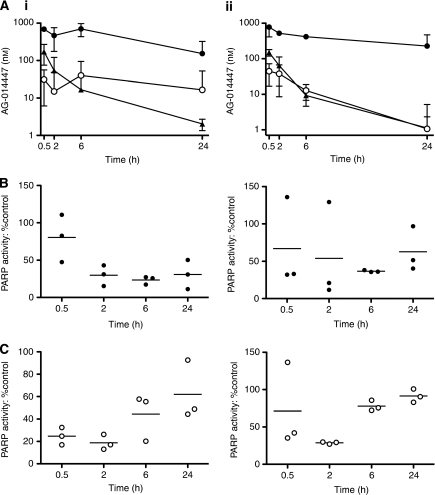
AG-014699 pharmacokinetics and PARP-1 inhibition in mouse brain and medulloblastoma xenograft. Following a single (i) or the last of four daily doses (ii) of AG-014699 (1 mg kg^−1^, i.p.), samples were taken and tissues harvested at the indicated times post-administration: (**A**) concentration of AG-014447 in plasma (solid triangle), brain (open circle) and D283Med xenograft homogenates (solid circle) from tumour-bearing mice. Corresponding PARP-1 activity in D283Med xenograft homogenates (**B**) and the brain (**C**) is shown. Concentrations of AG-014447 are given as the mean (±95% CI) from three tumour-bearing mice per time point; PARP activity measurements are given as individual values with the horizontal lines indicating the mean value.

After the first dose of AG-014699 (Figure 3Ai), a peak plasma concentration of 56±13 ng ml^−1^ (172±39 *μ*M) AG-014447 was detected at the earliest time point after injection. Thereafter, the levels decreased rapidly, such that at 24 h they were below the level of quantitation.

Levels in the tumour were higher than that in the plasma at all time points (Figure 3A), for example, 230–1510 nM in the tumour compared with 131–209 nM in plasma at 30 min. There was also significant and prolonged retention within the tumour, such that at 24 h after injection, levels of between 74 and 196 nM were still detectable. Surprisingly, given its physical and chemical properties, significant levels of AG-014447 were also detected in the brain tissue. Although these were initially lower than those in the plasma (20–40 nM at 30 min), there was some degree of retention such that at 24 h the levels were up to 10-fold higher than that in the plasma (1–30 nM in brain compared with 1.9–2.4 nM in plasma). After the fourth daily dose of AG-014699 (Figure 3Aii), peak plasma AG-014447 concentrations were similar to those following a single dose. Levels in the brain were only marginally greater than plasma levels at 6 h. However, high concentrations (639–932 nM) were retained in the tumour for the entire period. In the plasma, AUCs (26.4 and 21.9 *μ*mol l^−1^ min on days 1 and 4) and half-life (299 and 254 min) were similar to those reported previously (Thomas *et al*, 2007). The AUCs in the brain (37.0 and 14.7 *μ*mol l^−1^ min on days 1 and 4) were comparable to those in the plasma, whereas AUCs in tumour were substantially higher (582.0 and 509.6 *μ*mol l^−1^ min).

Consistent with the AG-014447 distribution data, PARP activity was suppressed in both brain and tumour tissue following administration of AG-014699. In the brain, PARP activity was reduced by around 75% for the first 2 h, recovering gradually thereafter, such that it was approximately 40% reduced at 24 h (Figure 3Bi). After the fourth daily dose, however, there was less suppression and more rapid recovery such that near-normal activity was detected at 24 h (Figure 3Bii). This presumably reflects the lower AUC of the drug in this tissue after the fourth dose. In contrast, in the tumour, inhibition of PARP activity was slightly delayed, reaching a nadir of around 75% reduction at 6 h post-injection, which was similar (barring obvious outlier data) after the first and fourth dose (Figure 3Ci and ii). The modest recovery at 24 h did appear greater after the fourth dose than the first, however.

### Efficacy of temozolomide with AG-014699 in human tumour xenografts

We examined the effect of AG-014699 on the anti-tumour activity of temozolomide in mice bearing established subcutaneous D425Med, D283Med or D384Med xenografts. Mice were treated daily for 5 days with either vehicle control alone, AG-014699 alone (1 mg kg^−1^ i.p.), TMZ alone (68 mg kg^−1^ p.o.) or the combination of TMZ (68 mg kg^−1^ p.o.)+AG-014699 (1 mg kg^−1^ i.p.) data are summarised in Table 2.

**Figure 4:**
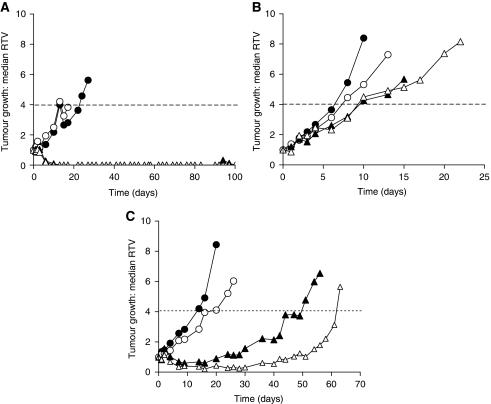
Enhancement of TMZ efficacy by AG-014699 in *in vivo* models of medulloblastoma. Growth of D425Med (**A**), D283Med (**B**) or D384Med (**C**) tumour xenografts over a 100-day period, following daily treatment for 5 days with vehicle control alone (solid circles), AG-014699 alone (1 mg kg^−1^, open circles), TMZ alone (68 mg kg^−1^, solid triangles) or TMZ (68 mg kg^−1^)+AG-014699 (1 mg kg^−1^) (open triangles). Dashed horizontal line corresponds to RTV4. Data are median of five mice per group until first humane killing owing to tumour burden.

At 1 mg kg^−1^ daily five times, AG-014699 alone did not cause any marked toxicity or affect tumour growth compared with vehicle-only controls. The D425Med cells grew relatively slowly (median time to RTV4=19 days) and, as expected from the *in vitro* data, were very responsive to temozolomide alone with complete tumour regressions seen in all mice (Figure 4A). These regressions were sustained for the designated experimental period (100 days) in two out of five mice. Co-administration of AG-014699 with temozolomide also resulted in complete tumour regressions in all mice, of which three out of five were sustained throughout the experiment. The MMR-defective D283Med xenografts grew very rapidly (median time to RTV4=7 days) and showed very little response to temozolomide alone (TGD of only 2 days) with no regressions observed in any mice (Figure 4B). In contrast to the pronounced *in vitro* sensitisation, co-administration of AG-014699 only increased this TGD to 2.5 days. The D384Med xenografts, proficient in both MGMT and MMR pathways of DNA repair, grew at an intermediate rate (median time to RTV4=16 days). Temozolomide alone caused a significant TGD (*P*=0.016), extending the time to RTV4 to 44.5 days (Figure 4C), and the combination with AG-014699 the time to RTV4 was 62 days. Thus, the temozolomide-induced TGD of 28.5 days was extended to 46 days by co-administration of AG-014699, equivalent to a 61% increase in efficacy; however, this was not quite significant (*P*=0.11 Mann–Whitney test). There was one transient complete response seen in both the temozolomide alone and the temozolomide+AG-014699 groups. Temozolomide alone caused a modest (5±4%), but statistically significant, weight loss relative to control (*P*=0.0006, unpaired *t*-test). AG-014699 was not toxic *per se* (1±1% body weight loss), but caused a modest, but significant, enhancement of temozolomide-induced body weight loss (10±6% weight loss: *P*=0.013, unpaired *t*-test).

## Discussion

In the work described here, we sought to address the need for new therapeutic approaches to improve outcome in medulloblastoma. Temozolomide shows good activity in adult glioblastomas (Mrugala and Chamberlain, 2008) and encouraging data are emerging from Phase I and II studies in paediatric intra-cranial malignancies, including medulloblastoma (Barone *et al*, 2006; Baruchel *et al*, 2006; Nicholson *et al*, 2007; Wang *et al*, 2009). We assessed the efficacy of temozolomide alone, and in combination with the PARP inhibitor AG-014699 in medulloblastoma, using three models (D384Med, D425Med and D283Med), which are genetically representative of the primary disease (Langdon *et al*, 2006). We also investigated pharmacokinetics, pharmacodynamics and toxicity of AG-014699, and show for the first time uptake into CNS and significant and sustained PARP inhibition in brain tissue.

We initially investigated in our models the status of molecular pathways implicated in the modulation of temozolomide sensitivity (i.e. DNA MMR and MGMT; Middlemas *et al*, 2000). Our cell lines modelled closely the diversity observed in primary medulloblastomas; D384Med cells were proficient for all proteins tested, indicating they reflect the vast majority of primary medulloblastomas, in which MMR deficits (observed in ∼10% of cases; Viana-Pereira *et al*, 2009) and MGMT hypermethylation (∼25% of cases; Lindsey *et al*, 2005) are relatively uncommon. Data for our other cell lines were consistent with their relevance as models of primary cases associated with MMR (D283Med) and MGMT (D425Med) deficiency. In subsequent efficacy testing, the MGMT-deficient D425Med cells were very sensitive to temozolomide both in cell cultures and as xenografts, being nearly 25 times more sensitive than the MGMT-proficient D384Med cells in culture, and showing complete tumour regressions in response to temozolomide alone in *in vivo* experiments. The sensitivity of D425Med to temozolomide alone may limit the usefulness of this cell line in assessing sensitisation by inhibition of PARP.

Previous studies in paediatric xenografts have also concluded that MGMT status is the major determinant of sensitivity to temozolomide, but that MMR defects also confer resistance (Middlemas *et al*, 2000). This was clearly illustrated in our panel by MMR-defective D283Med cells, which were nearly four times less sensitive to temozolomide than D384Med cells and xenografts derived from these cells showed barely any response to temozolomide therapy. In cell culture experiments, AG-014699, at a concentration that inhibited PARP by >95%, did not enhance temozolomide sensitivity in the MGMT-defective cells, but caused a striking 20-fold enhancement in the MMR-defective D283Med cells. These observations are in line with our previous observations in adult human cancer cell lines (Calabrese *et al*, 2004; Curtin *et al*, 2004; Thomas *et al*, 2007). These data reflect the molecular pathology of the cells and hence the relative contribution that O^6^-methylguanine and N^7^-methylguanine and N^3^-methyladenine (that are repaired by PARP-dependent processes) make to the overall cytotoxicity of temozolomide in the individual cell lines.

In the *in vivo* chemosensitisation studies, co-administration of AG-014699 caused an approximately 60% increase in temozolomide-induced TGD in the DNA repair protein-competent D384Med xenografts but, owing to the small sample number, the effect was not significant (*P*=0.11). In comparison, the D425Med tumours grew relatively slowly and responded well to temozolomide alone, with all mice showing complete tumour regressions, two of which were sustained until the termination of the experiment at 100 days. Xenografts from MGMT-deficient SW620 cells show similar sensitivity to temozolomide alone, but are sensitised further by AG14361 and AG-014699 (Calabrese *et al*, 2004; Thomas *et al*, 2007), most probably because of the vasoactivity of these PARP inhibitors (Ali *et al*, 2009). We had therefore expected to see potentiation of temozolomide anti-tumour activity in D425Med tumours by AG-014699. The number of complete regressions persisting to the end of the experiment was three out of five in the temozolomide+AG-014699 group, compared with two out of five in the temozolomide alone. On the basis of the small sample size, this cannot be considered significant, but is nevertheless encouraging. The lack of sensitisation by AG-014699 of the anti-tumour effect of temozolomide in MMR-defective D283Med xenografts was surprising, given the degree of potentiation seen *in vitro*. The lack of effect of AG-014699 in the D283Med xenografts was not due to PK/PD limitations, as accumulation of AG-0144447 and substantial PARP inhibition was shown in the tumours. MMR defects are observed in only a minority of medulloblastomas and the lack of synergy in this model does not undermine the rationale for combining PARP inhibitors with temozolomide. Thus, in summary, we propose that the D384Med model most faithfully reflects the clinical situation in the majority of medulloblastoma cases. The indication of chemosensitisation observed in this model is therefore encouraging and warrants further investigation in a wider panel of MMR- and MGMT-competent medulloblastoma models.

We have not previously determined the CNS penetration of AG-014447, the free drug of which AG-014699 is the phosphate salt, nor its inhibition of PARP activity in the brain. We saw clear evidence of AG-014447 penetration into brain tissue, and accompanying evidence of PARP inhibition, in mice with an intact BBB. PARP activity in the brain was higher than expected for a non-dividing tissue, with the activity being in the range 88–106 pmol PAR per mg protein per min. Although significantly lower than that detected in the associated tumour xenografts (1402–3236 pmol PAR per mg protein per min), it was comparable with the activity detected in normal mouse liver and kidney (98–106 pmol PAR per mg protein per min in the livers, 69–76 pmol PAR per mg protein per min in the kidneys, unpublished observations). Although concentrations of AG-014447 achieved in the brain (20–60 nM) were lower than were used for *in vitro* chemosensitisation, the inhibition of PARP activity over the 24-h period was 50–80%. This level of inhibition is in general agreement with the 80% inhibition of activity seen in permeabilised cells exposed to 100 nM AG-014699, and is sufficient to cause *in vivo* chemosensitisation in models of both adult and paediatric malignancies (Calabrese *et al*, 2004; Daniel *et al*, 2009). Importantly, the peak plasma level in mice treated with AG-014699 at 1 mg kg^−1^ was 42–68 ng ml^−1^, which is approximately 1/10th the concentration in patients treated with the recommended Phase II dose of 12 mg m^−2^ (473–675 ng ml^−1^). Therefore, higher drug levels and greater PARP inhibition should be achievable in the brains of patients treated with a safe dose of AG-014699. The observed accumulation of AG-014447 in xenografted medulloblastomas, which is consistent with our data for other tumour types coupled with a potentially compromised blood–brain tumour barrier suggests that drug accumulation in the tumours may be even higher. Further investigations in orthotopic or spontaneous transgenic models of medulloblastoma could provide useful insights in this regard, although the limitations of these models as representative of primary tumours and an intact BBB must also be taken into account.

Several PARP inhibitors have been shown to penetrate CNS tissue and to have a pharmacological effect, either in terms of an enhancement of the anti-tumour activity of temozolomide against intra-cranial tumours or reduction in focal ischaemia in a stroke model (Tentori *et al*, 2003; Hattori *et al*, 2004; Cheng *et al*, 2005; Donawho *et al*, 2007). Indeed, one of the indications of PARP inhibitors is in the reduction of brain tissue damage following stroke (Jagtap and Szabo, 2005). However, to the best of our knowledge, this is the first time that inhibition of PARP activity in the brains of mice treated with a PARP inhibitor has been shown.

In summary, the data we present here shows encouraging enhancement of temozolomide activity in pre-clinical cell line models of medulloblastoma, representative of the spectrum of sensitivity to single-agent temozolomide. Furthermore, following a non-toxic dose of AG-014699, good CNS penetration and correspondingly substantial inhibition of PARP activity in nervous tissue was observed in association with AG-014699 plasma levels 1/10th of those detected in patients treated with the recommended Phase II dose, and without significant toxicity. Further validation of these observations in a wider range of cell lines and in orthotopic models are required before a clinical evaluation of AG-014699 in combination with temozolomide for intra-cranial malignancies would be indicated.

## Figures and Tables

**Table 1:** Growth inhibition by temozolomide and sensitisation by AG-014699

**Cell line**	**GI_50_ temozolomide (*μ*M)**	**GI_50_ temozolomide+AG-014699 (*μ*M)**	**PF_50_**
D425Med	9.3±7.5^a^	13.3±3.2	0.7±0.4
D283Med	807±239	42±15^*^	19±4.7
D384Med	220±42	60±13^**^	3.8±1

Abbreviations: GI_50_=inhibition of growth by 50% PI_50_=potentiation factor_50_.

a Data are means and standard deviation of three independent experiments of the type shown in Figure 2.

^*^Significantly different from temozolomide alone (*P*=0.005, Student's two-tailed *t*-test).

^**^Significantly different from temozolomide alone (*P*=0.003, Student's two-tailed *t*-test).

**Table 2:** Toxicity and efficacy of TMZ and AG-014699 in medulloblastoma xenografts

**Xenograft**	**Treatment**	**Median nadir body weight (% starting weight)**	**Median time to RTV4 (days)**	**Tumour growth delay (days) (% enhancement)**	**Complete regressions**
D425Med	Control	98	19		0/5
	AG-014699	98	15.5		0/5
	TMZ	96	>100	>81	2/5
	Combination	95	>100	>81 (N/A)	3/5
					
D283Med	Control	98	7		0/5
	AG-014699	100	8	1	0/5
	TMZ	94	9	2	0/5
	Combination	91	9.5	2.5 (25%)	0/5
					
D384Med	Control	100	16		0/5
	AG-014699	96	20	4	0/5
	TMZ	97	44.5	28.5	1/5
	Combination	90	62	46 (61%)	1/5

Abbreviations: N/A=not applicable; RTV=relative tumour volume; TMZ=temozolomide; PI_50_=potentiation factor_50_.
